# Immune-Related Protein Interaction Network in Severe COVID-19 Patients toward the Identification of Key Proteins and Drug Repurposing

**DOI:** 10.3390/biom12050690

**Published:** 2022-05-11

**Authors:** Pakorn Sagulkoo, Apichat Suratanee, Kitiporn Plaimas

**Affiliations:** 1 Program in Bioinformatics and Computational Biology, Graduate School, Chulalongkorn University, Bangkok 10330, Thailand; pakorn.sagulkoo@cmu.ac.th; 2 Center of Biomedical Informatics, Department of Family Medicine, Faculty of Medicine, Chiang Mai University, Chiang Mai 50200, Thailand; 3 Department of Mathematics, Faculty of Applied Science, King Mongkut’s University of Technology North Bangkok, Bangkok 10800, Thailand; apichat.s@sci.kmutnb.ac.th; 4 Intelligent and Nonlinear Dynamics Innovations Research Center, Science and Technology Research Institute, King Mongkut’s University of Technology North Bangkok, Bangkok 10800, Thailand; 5 Advance Virtual and Intelligent Computing (AVIC) Center, Department of Mathematics and Computer Science, Faculty of Science, Chulalongkorn University, Bangkok 10330, Thailand; 6 Omics Science and Bioinformatics Center, Faculty of Science, Chulalongkorn University, Bangkok 10330, Thailand

**Keywords:** severe COVID-19, immune system, network diffusion, protein-protein interaction network, drug repurposing

## Abstract

Coronavirus disease 2019 (COVID-19) is still an active global public health issue. Although vaccines and therapeutic options are available, some patients experience severe conditions and need critical care support. Hence, identifying key genes or proteins involved in immune-related severe COVID-19 is necessary to find or develop the targeted therapies. This study proposed a novel construction of an immune-related protein interaction network (IPIN) in severe cases with the use of a network diffusion technique on a human interactome network and transcriptomic data. Enrichment analysis revealed that the IPIN was mainly associated with antiviral, innate immune, apoptosis, cell division, and cell cycle regulation signaling pathways. Twenty-three proteins were identified as key proteins to find associated drugs. Finally, poly (I:C), mitomycin C, decitabine, gemcitabine, hydroxyurea, tamoxifen, and curcumin were the potential drugs interacting with the key proteins to heal severe COVID-19. In conclusion, IPIN can be a good representative network for the immune system that integrates the protein interaction network and transcriptomic data. Thus, the key proteins and target drugs in IPIN help to find a new treatment with the use of existing drugs to treat the disease apart from vaccination and conventional antiviral therapy.

## 1. Introduction

Coronavirus disease 2019 (COVID-19) has been spreading worldwide, despite several developed vaccines, still causing numerous cases. Moreover, most causes of death are from severe complications from the disease. Currently, the global statistical data from the World Health Organization (WHO) indicate that 476,374,234 and 6,108,976 cases are infected and dead, respectively (26 March 2022) [1]. COVID-19 is an infectious disease caused by severe acute respiratory syndrome coronavirus-2 (SARS-CoV-2), a positive-sense single-strand RNA (+ssRNA) virus [2]. SARS-CoV-2 is a betacoronavirus, classified in the Coronaviridae family [3]. Although SARS-CoV-2 incurs a well-known pandemic coronavirus infection in the present, for severe respiratory diseases resulting from the two coronavirus diseases, most known cases emerged before, in the last two decades. For example, SARS-CoV pneumonia occurred from November 2002 to August 2003 from Guangdong, China, spreading to 30 countries worldwide, having 8422 confirmed cases and 916 deaths [4]. Several epidemiolocal studies suggested that palm civets (Paguma larvata) in a market in Guangdong were the initial hosts for SAR-CoV infection before the emergence of human-to-human transmission [5]. Middle East Respiratory Syndrome (MERS) was first reported in Jeddah, Saudi Arabia, in June 2012. It then spread to many countries in the Arabian Peninsula and to some countries in North Africa, Western Europe, East Asia, Southeast Asia, and North America [6]. The disease is caused by MERS coronavirus (MERS-CoV), which has much evidence indicating that its hosts are camels [7]. Various reports show 2578 confirmed cases and 888 deaths from MERS [8,9]. The first case of COVID-19 was reported in Wuhan, China, in December 2019 [10]. The patient was diagnosed with severe pneumonia with an unknown cause [11], while the number of cases in Wuhan increased to 41 in January 2020. In the same month, the first evidence revealed human-human transmission and asymptomatic or pre-symptomatic transmission [12]. Afterwards, COVID-19 spread from China to Thailand, Singapore, Vietnam, Taiwan, Japan, South Korea, Nepal, and the United States [13]. On 11 February 2020, COVID-19 was declared a pandemic by the WHO [12].

As mentioned above, COVID-19 is caused by SARS-CoV-2, a +ssRNA virus classified as a betacoronavirus. SARS-CoV-2 genome sequence shares 79% of genes with SARS-CoV genome compared to MERS-CoV, which has only 53% similarity. Nonetheless, SARS-CoV-2 has a percent identity of more than 96% when compared to bat-SARS-like CoV (SL-CoV), suggesting that bats can be an initial host of COVID-19 [14]. SARS-CoV-2 genome also shares 85.5 to 92.4% of identity with pangolin coronavirus genomes, indicating that pangolins could be an initial host of the infection [15]. The genomic content of SARS-CoV-2 consists of 16 non-structural proteins (NPs), 4 structural proteins, and 9 putative accessory factors [16]. The 16 NPs contain open reading frame (ORF) 1a and 1b. There are three crucial NPs that play a vital role in SARS-CoV-2 replication and pathogenesis. For instance, papain-like protease (PLpro) and 3C-like protease (3CLpro) have functions to cleave the viral polyprotein translated from ORF1a and ORF1b into 16 NPs. RNA dependent RNA polymerase (RdRp) replicates the viral genome in host cells [17,18]. Structural proteins, composed of spike (S), envelope (E), membrane (M), and nucleocapsid (N) protein, play a significant role as a viral genome protector and virulent factors used for virus entry [19]. The putative accessory factors are encoded from ORF3b, ORF6, ORF7a and ORF8. Their roles are not well understood, although some studies revealed that they were involved in interferon antagonism and impaired host immune response [20].

The pathogenesis of COVID-19 occurs when the virus enters a host respiratory epithelial cell using an S protein primed by transmembrane serine protease 2 (TMPRSS2) binding with a host membrane receptor, such as angiotensin-converting enzyme 2 (ACE2) receptor [21,22]. Meanwhile, SARS-CoV-2 also binds with Toll-like receptors (TLR) 4 and 8, causing innate immune response that will be described in further detail [23]. After entering the host cell, the viral genetic material is replicated to copied viral genomes and translated to essential viral proteins in the host cytoplasm. The copied viral genomes are assembled with the translated structural proteins to form the mature viral particles. Then, the replicated virions are released from the infected host cell and enter other non-infected host cells [24]. During the viral replication, some viral components become pathogen-associated molecular patterns (PAMPs) while the infected host cells express endogenous damage-associated molecular patterns (DAMPs). These molecules are recognized by pattern recognition receptors (PRRs) in the host cells such as TLR-3, 7, and 8, retinoic acid-inducible gene 1 (RIG-1)-like receptors (RLRs)/melanoma differentiation-associated gene 5 (MDA5), and NOD-like receptors (NLRs) [25]. The interaction between PAMPs including DAMPs and PRRs in the infected host cells activates the host innate immune response by promoting the production of antiviral and proinflammatory cytokines; for example, interferon α (IFN-α), IFN- β, IFN-γ, interleukin 1β (IL-1β), IL-6, IL-12, IL-18, IL-33, and tumor necrosis factor α (TNF-α) [26]. Moreover, PAMPs and DAMPs’ interaction with PRRs releases nuclear factor κB (NF-κB) [27]. NF-κB from the infected host cell stimulates innate immune cells such as dendritic cells (DCs), monocytes, macrophages, neutrophils, and natural killer (NK) cells to secrete further proinflammatory cytokines [27,28]. As a result, the uncontrolled proinflammatory cytokines are excessively released from both immune and respiratory epithelial cells, leading to collateral tissue damage. This phenomenon is called hyperinflammation or a cytokine storm, the most common fatal complication in COVID-19 [29].

Pathophysiology of severe COVID-19 is initiated when cytokine storm injures lung epithelial and endothelial cell damage and induces apoptosis, resulting in increased pulmonary vascular permeability [30]. The plasma is then leaked from the capillary to the alveolar space. Consequently, the gas exchange defect occurs, leading to acute respiratory distress syndrome (ARDS). Patients with ARDS will have progressive dyspnea, hypoxia and require ventilation support and intensive care [31]. Unfortunately, the excessive proinflammatory cytokines also affect other organs, such as the gastrointestinal tract, cardiovascular system, brain, liver, and kidney [32]. As a result, the patients will progress the signs and symptoms of multiple organ injuries; for instance, nausea, vomiting, diarrhea, hemodynamic instability, alterative mental status, heart tissue damage, elevated liver enzyme, and creatinine rising [33]. Cytokine storms not only directly injure several organs but also generate organ infarction due to increased thromboembolic phenomena [34]. In addition, disseminated intravascular coagulation (DIC) can arise in severe COVID-19 because of the persistent coagulation factor and platelet consumption, inducing further multiple organ injury [35]. Severe COVID-19 patients usually die from multiple organ dysfunction [36]. Cytokine storms can also provoke other serious conditions, such as secondary hemophagocytic lymphohistiocytosis (sHLH) and macrophage activation syndrome (MAS), characterized by monocytes and macrophages engulfing erythrocytes, platelets, immune cells, and other host cells. Hence, both sHLH and MAS can promote more collateral tissue damage, worsening multiple organ dysfunction [37].

The current treatment trends in COVID-19 are using vaccination to prevent the disease and prescribing antiviral agents to infected people [38,39,40,41]. Although COVID-19 susceptibility and severity can be improved by using well-developed vaccines and effective antiviral therapies, some patients still progress the disease to the cytokine storm and other severe complications. COVID-19-associated cytokine storms can be treated with intravenous corticosteroid. Several systemic reviews and meta-analyses have indicated that systemic corticosteroids can improve critically ill COVID-19 patients [42,43,44,45,46]. However, systemic corticosteroid provides many unexpected side effects, such as hyperglycemia, adrenal suppression, and increased secondary bacterial infection [47,48,49]. Therefore, finding novel target treatments in severe COVID-19 instead of conventional medication is necessary for more effective treatment and fewer side effects. Drug repositioning is another technique to discover an existing drug to treat a disease. Systems biology and network analysis have been directly applied to identify key genes or proteins [50,51,52,53], drug–gene or drug–protein interactions [54,55], and drug–disease associations [56,57]. Structural bioinformatics is the main task at the molecular structure level to identify possibilities of compound targeting, such as the study of inverse docking fingerprints in drug repurposing for SAR-CoV-2 [58]. Modern biopharmaceutical approaches, such as large biomolecules like antibodies (immunoglobulins) and plasma, are of interest to treat COVID-19. However, their roles in clinical trials are currently being studied to treat severe cases.

In the precision medicine and data science era, bioinformatics and systems biology are central in molecular medicine and targeted therapy. Network biology is a powerful tool to identify key genes and targeted drugs involved in many diseases by using topological analysis and network diffusion algorithms. In addition, protein–protein interaction network analysis is usually used to find hub and bottleneck proteins [59,60,61], to infer protein functions [62,63,64], find gene–disease [65,66,67] and disease–disease associations [68,69]. Various centrality calculations, such as degree, betweenness, closeness, and eigenvector, play an important role in biological network analysis. Several systems biological studies have performed these centralities to identify key genes and gene prioritization in the disease-related networks [53,70,71,72]. Nonetheless, analyses of importance and difference of the centralities in biological roles are still needed for further investigation.

There are two methods for extracting a specific subnetwork: the neighborhood and diffusion approaches. Neighborhood method is a traditional technique performed widely in many biological network studies [73,74,75,76,77]. Furthermore, there are several COVID-19 network studies constructing protein–protein interaction networks and selecting a group of related proteins using this method [50,51,54,55,78,79,80]. This method extracts subnetworks by considering the nodes with the low shortest path with disease-associated genes or seed nodes [81]. The benefits of neighborhood-based subnetwork construction are that the approach is user-friendly and has fast running time. However, some neighbor nodes in the networks are not disease-associated genes, causing topological changes and missing identification of key nodes. Therefore, the diffusion-based method is preferred to build specific protein–protein interaction subnetworks, such as immune, inflammation, and viral–host interaction network, with lower false-positive disease-related nodes, although it is time consuming and requires coding skills. Network diffusion is the method used to predict novel disease-associated genes based on known disease-associated genes via considering the diffusion or probability scores from iterative running algorithms at time stable. Nodes with high diffusion scores are inferred as theoretical disease-related genes [82].

Moreover, there have been no COVID-19 studies involved in immune-related biological networks using the network diffusion method. As a result, we proposed this method to construct an immune-related protein interaction network (IPIN). A network diffusion method named Laplacian heat diffusion (LHD) algorithm on a human interactome network was conducted in this study to construct an IPIN in severe COVID-19 patients. Key immune-related proteins in the network were identified using several centralities and ranking score measurement. Additionally, drug repurposing was also performed to find target medication to those key proteins. This study aims to discover candidate target drugs to treat severe COVID-19 at clinical levels.

## 2. Materials and Methods

**Figure 1:**
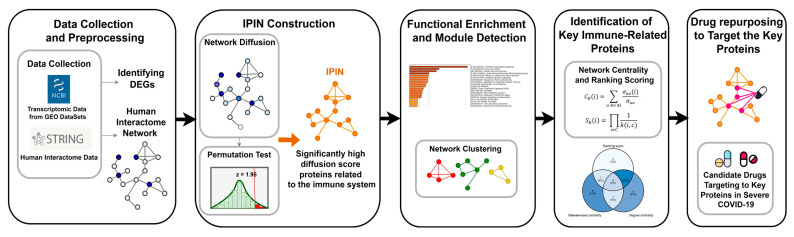
Summary of the process to identify the key proteins and drug repurposing in the severe COVID-19 based on an immune-related protein interaction network (IPIN). Circles represent protein nodes in a protein-protein interaction network. Dark blue circles are nodes of the DEGs’ proteins. Light blue circles are nodes of proteins having diffuse scores. Orange circles represent high diffusion score proteins for IPIN. Red, green, and yellow circles are proteins in different modules. Pink circles are target proteins of an existing drug.

The summary of materials and methods used in our study is illustrated as a diagram in Figure 1. First, differentially expressed genes (DEGs) of the transcriptomic data downloaded from GEO DataSets were mapped with the human interactome data from STRING v11.0 database [83] for forming the seed nodes. Second, construction of a human protein interactome network obtained from STRING database [83]. Third, the Laplacian heat diffusion (LHD) algorithm operated network diffusion. Fourth, a permutation test filtered out false-positive high diffusion score nodes. Fifth, significantly high diffuse score nodes were used to construct the IPIN. Metascape [84] was also performed for the functional enrichment analysis of the network. Sixth, Molecular Complex Detection (MCODE) and the Markov Clustering (MCL) algorithm were conducted to find IPIN modules. Next, the centralities and the ranking score were calculated to identify the key immune-related proteins. Finally, candidate drugs targeting the key proteins were discovered by chemical and drug databases searching for drug–gene and drug–protein interactions.

### 2.1. Data Collection and Preprocessing

#### 2.1.1. Human Protein Interactome

The human protein interactome data, containing 19,566 proteins (or nodes) and 11,938,498 interactions, was obtained from STRING v11.0 database (https://string-db.org/ accessed on 20 November 2021) [83]. R package ‘dplyr’ [85] was used to manipulate the data. The interactions with a combined score between 900 and 999 were included. R package ‘igraph’ [86] was conducted to eliminate isolated nodes and multiple edges. As a result, the rest of the interactome had 11,334 proteins and 123,263 interactions. The combined scores between 900 and 999 were changed to a weighted score by rescaling into the range of [0.01, 1].

#### 2.1.2. Transcriptomic Data

The collected data was from Gill et al.’s study (2020) in GEO DataSets (GSE154998) (https://www.ncbi.nlm.nih.gov/geo/ accessed on 20 November 2021) [87]. The researchers collected leukocytes samples from COVID-19 cases and controls in an intensive care unit (ICU) to perform transcriptomic profiles by RNA sequencing (RNA-seq) method. The total sample size was 14, with 7 samples being COVID-19 cases and the rest being controls. The SARS-CoV-2-positive cases were confirmed using reverse transcription-polymerase chain reaction (RT-PCR) method. In addition, genes from RNA-seq data having the false discovery rate (*FDR*) < 0.05 and log2fold change (*log2 FC*) > 1.5 were differentially expressed genes (DEGs). Hence, there were 224 genes meeting the criterion (Table S1 in Supplementary Materials). The DEGs then were mapped with the protein list in the protein–protein interaction network of the STRING v11.0 database by using Ensembl ID joining [83]. Thus, there were 189 Ensembl protein IDs (Table S2 in Supplementary Materials).

With the use of network diffusion, the seed nodes were prepared from mapping between these 189 Ensembl IDs (the immune-related proteins from DEGs) and the human interactome data (in Section 2.1.1). This resulted in 141 seed nodes (Table S3 in Supplementary Materials).

### 2.2. IPIN Construction with Network Diffusion

#### 2.2.1. LHD Algorithm

This study used LHD algorithm to operate human interactome network propagation. In many studies, LHD is one of the most common network diffusion algorithms used to infer disease-associated genes or proteins [88,89]. Given a network called *G*, let *W* be a weight adjacency matrix of network *G* calculated from rescaling the combined scores and *D* be a diagonal matrix whose values are a degree of each node arranged diagonally. Laplacian matrix (*L*) was calculated from *D*−*W*. An initial diffusion vector ($H_{0}$) of all nodes is conducted normally by setting the initial heat diffusion score ($h_{0}$) to each node. In general, the initial heat scores ($h_{0}$) are set as 1/*n* where *n* is the number of seed nodes related to the subject of interest (immune-related proteins as seeds in our case) while the other nodes are set as 0. The diffusion vector at time *t* ($H_{t}$) was updated based on the previous diffusion vector at time *t−1* ($H_{t-1}$) according to this equation:

(1) $$H_{t}=H_{t-1}\times e^{-Lt}$$

where *e* is defined as Euler’s number (≈2.1828). The network diffusion was iterated based on Equation (1) until two consecutive diffusion vectors were relatively similar. In our case, the relative similarity is met if $║H_{t}-H_{t-1}║$ < 10^−6^, then the diffusion becomes stable. The latest diffusion scores after the stability are used as an indicator of the relevant scores to the seed nodes. In another word, a node with a high diffusion score was strongly associated with seed nodes.

In our cases, the initial diffusion vector ($H_{0}$) contained the initial scores of 11,334 protein nodes in the whole protein–protein interaction network. The initial scores for 141 seed nodes were set as 1/141 while other protein nodes are set as 0. After the network propagation was stable, the final scores were then used for further permutation test analysis. In this study, LHD algorithm was carried out by using R package ‘diffusr’ [90]. The parameters in the package were well-established using the default setting.

#### 2.2.2. Permutation Test

When LHD algorithm finishes the network propagation, in theory, the nodes with the high diffusion score are strongly associated with the disease-associated proteins. However, some nodes receiving the high diffusion score can be false-positive. This false-positive result can occur because some factors such as the topological structure of network *G* can provide a high diffusion score apart from the actual association with the seed nodes. Therefore, a permutation test should be conducted to filter out nodes with the false-positive high diffusion score. The permutation test measures whether nodes have a high diffusion score due to statistical significance or chance. The test was operated by assigning 1000 different sets of the initial seeds into the human interactome network for LHD algorithm. Before running the algorithm in each set, 141 seed nodes were randomly assigned, independent of DEGs, from 11,334 nodes. Hence, the sets of seed nodes in the original set and in the 1000 sets were different. The z-score of the diffusion score of node *n* (Z(n)) was calculated according to this equation:

(2) $$Z\left(n\right)=\frac{h\left(n\right)-\bar{X}\left(n\right)}{SD\left(n\right)}\;,$$

where *h(n)* is defined as the diffusion score of node *n* in the original set, $\bar{X}\left(n\right)$ and SD(n) are the mean and standard deviation, respectively, of diffusion score of node *n* in the original set and 1000 permutation sets. A node with a z-score more than 1.96 (*p*-value < 0.05) had the true-positive high diffusion score and was selected for the IPIN construction. Table S4 in Supplementary Materials shows the original diffuse scores including with the mean and standard deviation calculated from the diffusion score in the original set and 1000 permutation sets in each node in the whole protein–protein interaction network.

#### 2.2.3. IPIN Construction

After the network diffusion by LHD algorithm and the validation of the high diffuse score nodes by permutation testing were performed, 154 nodes with significant diffusion scores were obtained (Table S5 in Supplementary Materials). The filtered significant nodes from the permutation test were mapped with the STRING human interactome network. The largest component of the significantly high diffusion score nodes consisting of 97 nodes and 778 interactions was selected as our immune-related protein interaction network or IPIN for further functional enrichment analysis, topological analysis, and centrality measurement. The construction of the IPIN was based on leukocytes or white blood cells’ transcriptome data. Leukocytes are the cells that play an important role in pathogen defense mechanisms or immunity. The gene expression of leukocytes during the infection indicates the host’s immune status against the pathogens. Thus, this IPIN was constructed to represent a core host immune system against the severe COVID-19. The edge list information for this IPIN is also provided in Table S14 in Supplementary Materials.

### 2.3. Topological Analysis, Network Centrality, and Ranking Scores

R package ‘igraph’ [86] was performed to calculate global and local topological parameters and network centrality [86]. The global topological parameters such as the number of nodes (*N*), the number of edges (*M*), average degree (<*k*>), diameter (*D*), mean shortest path length (*mspl*), and average clustering coefficient (*acc*) were computed. Furthermore, degree distribution and a clustering coefficient versus degree curve were plotted to evaluate the scale-free network properties. Several local parameters and centrality measurements such as degree centrality ($C_{D}$), betweenness centrality ($C_{B}$), closeness centrality ($C_{C}$), and eigenvector centrality ($C_{E}$) were calculated to find the essential nodes in the network. A node with more than 90th percentile centrality value was listed and its functional importance in the network and the disease was discussed. These high-value nodes in the four centrality sets were plotted using an upset plot from R package ‘UpSetR’ [91] to find the node overlapping in each centrality.

Degree centrality is the number of adjacent nodes having the interaction with interested node *i*, according to this equation

(3) $$C_{D}\left(i\right)=\underset{j}{\sum }A_{ij}\;,\;$$

where *A_ij_* is a value of a non-weight adjacency matrix (*A*) of node *i* and *j*, respectively. In network biology, the high-degree proteins are hub nodes and play an important role in the network function [92]. Therefore, numerous medications are designed for targeting the hub nodes.

Betweenness centrality is the summation of the ratio of the shortest path between node *u* and *v* that passes through node *i*. The betweenness centrality equation is

(4) $$C_{B}\left(i\right)=\underset{u\;\neq v\;\neq i}{\sum }\frac{\sigma_{uv}\left(i\right)}{\sigma_{uv}}\;,$$

where $\sigma_{uv}$ is a total number of the shortest path between node *u* and *v* and $\sigma_{uv}\left(i\right)$ is the number of the shortest path between node *u* and *v* that pass through node *i*. The high-betweenness proteins indicate the bottleneck property, forming the bridges controlling the flow of information in the network. Interruption of the bottlenecks by several targeted therapies can result in network function destruction in many diseases, improving the disease outcomes [92,93,94,95].

Closeness centrality is the summation of inverse shortest path distance between node *i* and all other nodes in the network. The closeness centrality equation is shown as

(5) $$C_{C}\left(i\right)=\frac{N-1}{\sum_{j=1,j\neq i}^{N}d\left(i,j\right)}\;,$$

where *N* is the total number of nodes in the network and d(i,j) is the shortest path length between node *i* and node *j*. Some studies used closeness centrality to find essential genes and proteins in several biological problems [96,97].

Eigenvector centrality is the extended form of degree centrality that focuses on the global high-degree nodes more than the local high-degree nodes. Eigenvector centrality has the assumption that the essential nodes should connect to the other important nodes. Therefore, nodes with high eigenvector centrality have high degree centrality, and their neighbored nodes also have a high degree value. The equation is demonstrated as

(6) $$C_{E}\left(i\right)=\frac{1}{\lambda }\;\underset{j}{\sum }A_{ij}C_{E}\left(j\right)\;,$$

where *λ* is the largest eigenvalue of *A*. Eigenvector centrality is applied to analyze many connectome studies in neurological diseases [98,99,100,101].

As mentioned above, all four centralities play a vital role in discovering essential genes or proteins. Moreover, numerous traditional network biology studies usually use degree and betweenness centrality to find essential genes and proteins. Closeness and eigenvector are occasionally applied to find vital genes and proteins. Therefore, a node with a high value of all centralities was a key protein in the network. The ranking score (*S_R_*) was applied to rank the nodes based on the centralities. Let *C* be a set of all centralities and *c* represent a centrality measure in *C*. A ranking score of any node *i* ($S_{R}\left(i\right)$) was calculated based on the reciprocal of the product of a ranking position of node *i* in each centrality *c* (k(i,c)), according to this equation

(7) $$S_{R}\left(i\right)=\underset{c\epsilon C}{\prod }\frac{1}{k\left(i,c\right)}.$$

Nodes with high-ranking scores greater than the 90th percentile were considered as key proteins in the IPIN network. In addition, nodes with degree and betweenness centrality greater than the 90th percentile were used to compare the ranking scoring nodes.

### 2.4. Functional Enrichment Analysis and Network Clustering

Metascape (https://metascape.org/gp/index.html#/main/step1 accessed on 10 December 2021) [84] was conducted for functional enrichment analysis in the largest component network according to the six terms: Gene ontology biological process (GO-BP) [102], Kyoto Encyclopedia of Genes and Genomes (KEGG) pathways [103], REACTOME pathways [104], WikiPathways [105], Canonical pathways [106], and CORUM pathway [107]. Moreover, the Molecular Complex Detection (MCODE) algorithm [108] in Metascape was operated to cluster the enrichment terms into large groups to find the more common biological terms. The Markov Clustering (MCL) algorithm [109] was also used in the STRING v11.0 database to explore the network communities in the IPIN. The inflation parameter of MCL was set to 1.5. Functional enrichment analysis in each module was also conducted in STRING v11.0 database using GO-BP, REACTOME pathways, KEGG pathways, and WikiPathways term. In addition, the clusters were visualized by using STRING v11.0.

### 2.5. Detection of Potential Drugs for Drug Repurposing

The key proteins, having a ranking score, degree, or betweenness centrality above the 90th percentile, were used as the input to find drug–gene and drug–protein interactions from DrugBank database (https://go.drugbank.com/ accessed on 15 December 2021) [110], Therapeutic Target Database (TTD) (http://db.idrblab.net/ttd/ accessed on 15 December 2021) [111], Comparative Toxicogenomics Databases (CTD) (http://ctdbase.org/ accessed on 15 December 2021) [112], and GeneCards (https://www.genecards.org/ accessed on 15 December 2021) [113]. Drugs which have United States Food and Drug Administration (FDA) approval and evidence of interactions with the key genes or proteins were selected. The STITCH v5.0 database (http://stitch.embl.de/ accessed on 18 December 2021) [114], containing drug–protein interaction information, was performed to confirm the chosen drugs. A confidence score of the interaction in the STITCH database was used to find suitable drug–protein interactions. The confidence score is the probability value calculated based on both experimental and computational evidence such as text mining, high-throughput experiments, co-expression and gene fusion data, and information from other databases. A drug with a confidence score of more than 0.9 was considered a candidate drug having efficiency for severe COVID-19 treatment.

## 3. Results

### 3.1. IPIN Construction and Topological Properties

**Figure 2:**
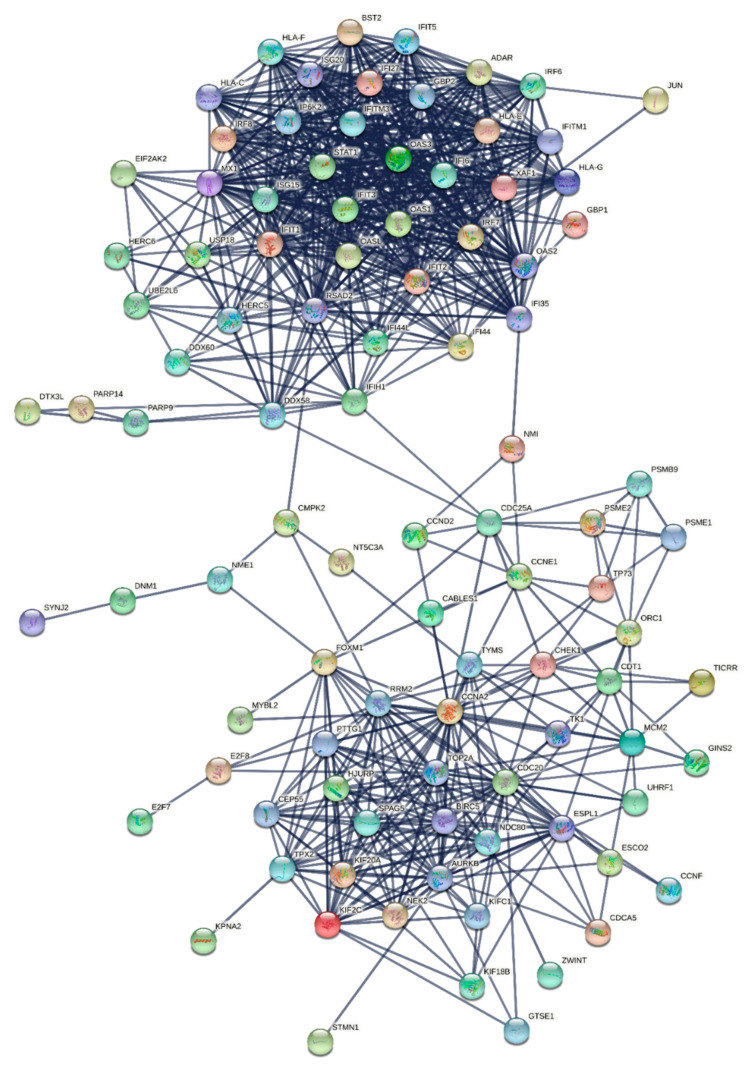
The largest component of the IPIN constructed from the mapping between the nodes with the significant diffusion scores and the human interactome network. The network consists of 97 nodes and 778 interactions.

The immune-related protein interaction network, known as IPIN, was obtained after the network diffusion and the validation of the high diffuse score nodes by permutation test. This network consisted of 97 nodes and 778 interactions as shown in Figure 2. In addition, the STRING reports showed that the average node degree and the expected edges are 16 and 50, respectively.

**Figure 3:**
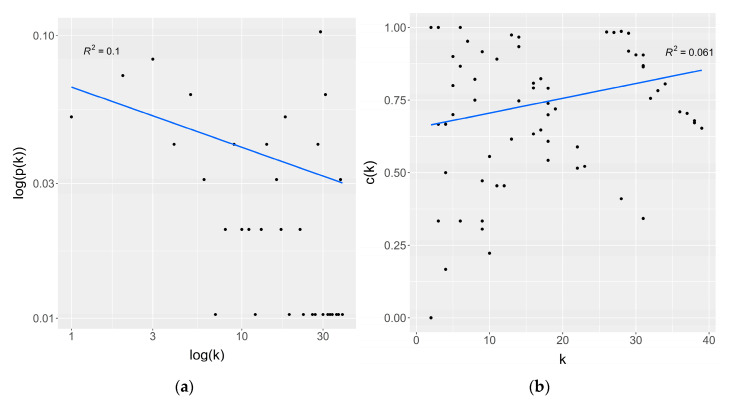
Topological analysis of the IPIN. (**a**) Degree distribution plot. (**b**) Clustering coefficient versus degree plot. *k* denotes the degree; p(*k*) denotes the probability of degree *k*; c(*k*) denotes the clustering coefficient of a node that has degree *k*.

Table 1 summarizes the global topological parameters of the IPIN. The average degree and diameter of the IPIN are 16.04 and 7, respectively. The network has the small-world effect because it provided the low mean shortest path length (*mspl* = 3.01) but the high average clustering coefficient (*acc* = 0.74). These behaviors are concordant with other biological networks. Local topological parameters in each node in the network, for example, clustering coefficient and degree, are summarized in Table S6 in Supplementary Materials. To find the scale-free property, a degree distribution was plotted to prove the power law, as shown in Figure 3a. Furthermore, a clustering coefficient versus degree plot is illustrated in Figure 3b. The degree distribution plot reveals that it does not follow the power-law distribution because it provides a low correlation (*R^2^* = 0.1). This appearance is explained by the IPIN is a real-world network extracted from the human interactome network. Thus, the power-law properties can be disrupted due to the subnetwork construction. Nevertheless, the clustering coefficient versus degree plot shows the independence between the clustering coefficient and degree (*R^2^* = 0.061). The independent relation between clustering coefficient and degree is found in random and scale-free networks [115]. Analysis in the STRING revealed that the IPIN’s protein–protein interaction (PPI) enrichment *p*-value is significant (*p*-value < 10^−16^), indicating that the proteins have interactions with each other more than by chance. Thus, the interactions in the IPIN were more significant than random interactions. The IPIN was less likely to be a random network even though there is no suitable reason to explain the scale-free properties of the IPIN.

### 3.2. Functional Enrichment and Module Identification in IPIN

**Figure 4:**
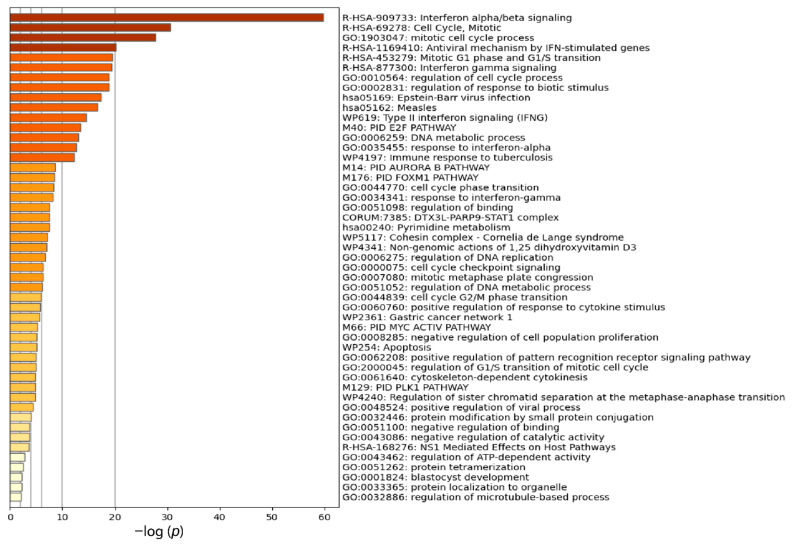
The bar graph represents the enrichment terms analyzed from the IPIN at a significant level (*p*-value < 0.01). Each enrichment term is colored based on the significance level.

The functional enrichment analysis was performed using a hypergeometric test and Benjamini–Hochberg statistical correction algorithm in Metascape [84], which integrates six biological and pathway enrichment terms: GO-BP [102], KEGG pathways [103], REACTOME pathways [104], WikiPathways [105], Canonical pathways [106], and CORUM pathway [107]. The results revealed that the terms were primarily involved in immune pathways, cell division, nucleotide metabolisms, and protein processing, as shown in Figure 4. The immune pathways were associated with innate immune response and antiviral signaling pathways such as type I and II IFN (IFN-I and IFN-II) and IFN-stimulated genes (ISGs). IFN-I mainly comprises of IFN-α and IFN-β, while IFN-γ is a component in IFN-II. The cell division terms were relevant to mitotic cell cycle process, mitotic metaphase, regulation of cell cycle, phase transition of cell cycle checkpoint at G1/S and G2/M, cytoskeleton-dependent cytokinesis, and regulation of sister chromatid separation. Moreover, nucleic metabolic pathways, such as pyrimidine metabolism, DNA metabolic process, and regulation of DNA replication, were identified. Apoptosis was also found from the enrichment analysis. Other enrichment terms were protein and enzyme processing, such as protein modification by small protein conjugation, negative regulation of catalytic activity, protein tetramerization, and protein localization to organelle.

**Figure 5:**
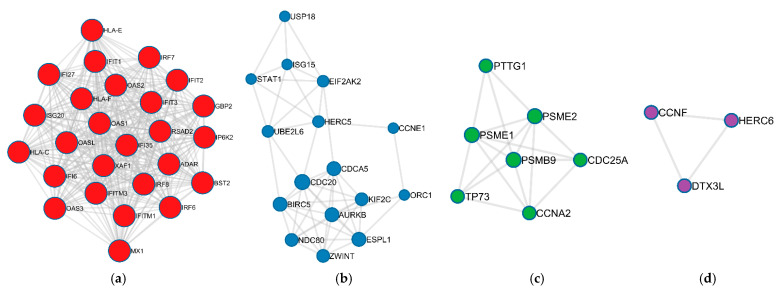
Module detection from the IPIN using MCODE algorithm in Metascape [84]. Circles represent protein nodes. Nodes in each subgraph are colored differently for different modules. The size of nodes represents their connectivity in the modules. (**a**) MCODE1 had 26 nodes (marked as red nodes) and 320 edges. (**b**) MCODE2 had 16 nodes (marked as blue nodes) and 49 edges. (**c**) MCODE3 had 7 nodes (marked as green nodes) and 17 edges. (**d**) MCODE4 had 3 nodes (marked as purple nodes) and 3 edges.

In addition, MCODE algorithm clustered the functional enrichment terms into four modules, MCODE1, MCODE2, MCODE3, and MCODE4, as shown in Figure 5. For instance, MCODE1 (Figure 5a) represented the biological term related to innate immune and proinflammatory cytokine signaling pathways: IFN-α and IFN-β. MCODE2 (Figure 5b) was associated with mitotic cell division, cell damage detection, and chromosome segregation. MCODE3 (Figure 5c) was involved in cell cycle checkpoint and cell cycle signaling pathways, and MCODE4 (Figure 5d) was enriched with protein processing and antigen presentation. Table 2 shows the enrichment analysis results from the MCODE algorithm.

**Figure 6:**
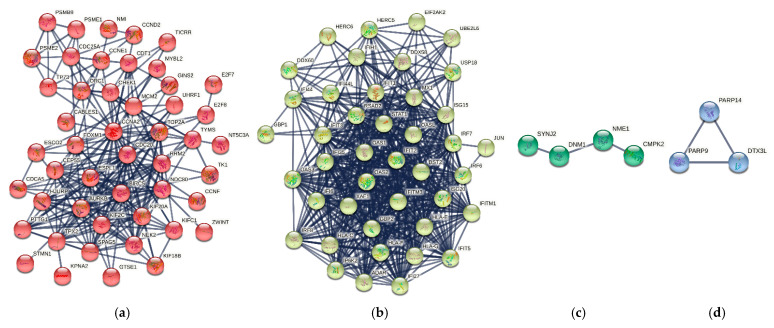
Module detection from the IPIN using MCL algorithm in STRING v11.0. Circles with different colors represent proteins in different clusters. (**a**) MCL1 had 48 nodes (marked as red) and 234 edges. (**b**) MCL2 had 42 nodes (marked as yellow) and 528 edges. (**c**) MCL3 had 4 nodes (marked as green) and 3 edges. (**d**) MCL4 had 3 nodes (marked as blue) and 3 edges.

Network clustering of the IPIN by MCL algorithm provided four modules: MCL1, MCL2, MCL3, and MCL4. Figure 6 demonstrates the MCL modules of the IPIN. Further detail about the clusters is described in Table S7 in Supplementary Materials. The result from functional enrichment analysis of the four modules revealed that the MCL1 (Figure 6a) was related to cell cycle regulation functions while MCL2 (Figure 6b) was mainly enriched in innate immune responses. Moreover, MCL3 (Figure 6c) had an association with nucleic acid metabolism. MCL4 (Figure 6d) mixed cell division and immune response terms. The details of enrichment results in each MCL module are listed in Table S8 in Supplementary Materials. The enrichments result was aggregable with the result from the MCODE analysis.

Functional enrichment and network clustering analysis revealed two large biological pathways: antiviral and innate immune response and cell division and cell cycle regulation (as shown in Figure 4, Figure 5 and Figure 6 and Table 2). The innate immune response found in the study mostly correlated with IFN signaling pathways. IFN can be separated into two groups: type I and II IFN (IFN-I and IFN-II). IFN-I cytokines, such as IFN-α and IFN-β, play an essential role in innate immunity to many viral infections [116]. They are released by the interaction between PAMPs or DAMPs and PRRs. The IFN-I activation controls innate immunity and increases viral clearance by stimulating various antiviral proteins, such as MX1, OASs, and ISGs [117,118,119,120,121]. IFN-I is also involved in NK cells, B lymphocyte, CD4^+^, and CD8^+^ T lymphocyte stimulation [121]. Nonetheless, IFN-I signaling is suppressed and delayed in coronavirus infections, such as SARS-CoV, MERS-CoV, and SARS-CoV-2, causing viral clearance dysfunction. Persistent viral replication causes the release of uncontrolled proinflammatory cytokines, resulting in the cytokine storm [116,122,123]. This phenomenon is also called paradoxical hyperinflammation. Although IFN-II, consisting of IFN-γ, has an immune function overlapping with IFN-I to stimulate the antiviral and innate immune response, it causes enhancement predominantly in antigen-presenting cells (APCs) [124]. Several studies have revealed that IFN-II exhaustion is usually found in severe COVID-19 patients, suggesting a vital role of IFN-I in the immunopathology of the disease [125,126,127]. As a result, IFN signaling enrichment found in the peripheral white blood cells can be from the compensating mechanism of immune cells for IFN suppression and delay in severe cases. However, IFN signaling predominance in this study can indicate the ongoing activation. Persistent IFN stimulation induces apoptosis of CD4+ T lymphocytes and causes lymphopenia. It also increases proinflammatory cytokine production [128,129,130]. Other evidence supports the notion that increased IFN-I in influenza viral infection can release excessive proinflammatory cytokines, resulting in respiratory epithelial apoptosis and severe pneumonia [131]. Because the IFN function in severe COVID-19 is complicated, the study of IFN roles in the disease should be more investigated.

Leukocyte proliferation is the immune defense mechanism responding to infections. However, cell division and cell cycle regulation can also participate in the pathophysiology of COVID-19-associated cytokine storms. In the RNA-seq study used to construct the IPIN, the patient data revealed that severe COVID-19 cases had elevated neutrophils more than lymphocytes [87]. An excessive number of neutrophils can cause increased production of proinflammatory cytokines. Hence, finding the key immune-related proteins in the cell cycle regulation is necessary to modulate the immune response in severe cases. Nucleic acid metabolism was also found in the enrichment analysis. Increased cell division also stimulates nucleic acid metabolism to produce DNA materials for chromosome segregation. Although cell division is found in leukocytes during the infection, several studies revealed that SARS-CoV could induce cell cycle arrest in host cell lines by using viral proteins interacting with cyclin and the cyclin-dependent kinase (CDK) complex [132,133,134]. Nevertheless, there is still no research about the relation between SARS-CoV-2 and leukocyte cell cycle regulation. Therefore, studying the cell cycle regulation of host cells in COVID-19 requires further investigation.

Another term found in the enrichment analysis was apoptosis or programmed cell death. Apoptosis in COVID-19 causes deleterious effects, leading to severe complications. For instance, lymphocyte apoptosis results in lymphopenia and delays adaptive immune response, increasing the cytokine storm risk. Many studies have found that B and T lymphocyte apoptosis is associated with severe COVID-19 [135,136,137]. Apoptosis is also found in respiratory epithelial and endothelial cells, causing the blood-air barrier defect. This event leads to ARDS progression. Therefore, apoptosis regulation in severe COVID-19 is essential to improve adaptive immunity and reduce fatality rates.

Furthermore, there was protein processing found in the IPIN enrichment analysis. Protein processing is post-translational modification occurring in the endoplasmic reticulum (ER) and Golgi apparatus. The immune cells during the infection have more active functions, such as cell proliferation and cytokine production. Hence, protein processing is highly expressed to play a role in these activities.

### 3.3. Key Immune-Related Proteins in the IPIN

#### 3.3.1. Important Immune-Related Proteins with Node Centralities

Network centrality aspects such as degree, betweenness, closeness, and eigenvector centralities of each node in the IPIN are illustrated in Table S6 in Supplementary Materials. Nodes with degree, betweenness, closeness, or eigenvector centralities above the 90th percentile are shown in Tables S9–S12 in Supplementary Materials, respectively. From the tables, 15 nodes have large degree values, and 10 nodes have high betweenness values. In addition, 11 and 10 nodes have high closeness and eigenvector scores, respectively. These nodes are proteins playing a role in antiviral, innate immune, apoptosis, and cell cycle regulation signaling pathways. The function of each protein including high centrality predominance is displayed in Table 3.

From the tables, we found that nodes with high betweenness values were primarily involved in cell cycle regulation (6 of 10 nodes), while nodes with high degree and eigenvector values were mainly related to antiviral and innate immune signaling pathways (13 of 15 nodes and 9 of 10 nodes with high degree and eigenvector values, respectively). In addition, we discovered that the high closeness nodes had a similar proportion between immune signaling and cell proliferation, explaining that 6 of 11 nodes were involved in the innate immune response. At the same time, the rest of the nodes had a function associated with cell cycle regulation.

**Figure 7:**
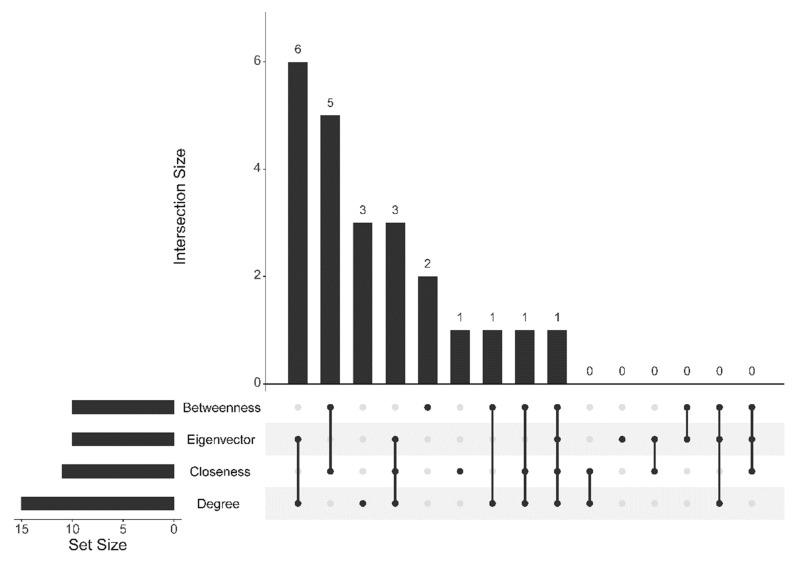
Upset plot of nodes with high values in the four centralities: degree, betweenness, closeness, and eigenvector centrality.

The upset plot In Figure 7 shows that all nodes with high eigenvector values are in the high degree nodes. That means the high-eigenvector nodes form a subset of the high-degree nodes. Furthermore, the high-betweenness nodes share the node members mainly with the high-closeness nodes (the intersection size is calculated from 5 + 1 = 6). There is one node shared in all centralities (RSAD2). The degree, betweenness, and closeness centrality provide unique nodes that do not intersect with other centralities. The numbers of unique nodes in degree, betweenness, and closeness centrality are 3, 2, and 1, respectively.

#### 3.3.2. Important Immune-Related Proteins with the Ranking Scores

As shown in Table 4, there are 10 nodes with a ranking score above the 90th percentile. Among them, eight nodes have functions relevant to innate immune response and antiviral activity: IFIT1, IFIT2, IFIT3, IRF7, ISG15, MX1, RSAD2, and STAT1. Interestingly, these immune nodes participate in IFN signaling pathways. The signaling pathway is usually activated when viral infections invade the hosts [138]. Stimulated IFNs increase the production of antiviral proteins, for example, ISG15 and MX1 [139,140]. In addition, the rest of the nodes, such as CDC25A and CCNA2, are involved in cell proliferation and cell cycle regulation. During infection, the immune system increases leukocyte proliferation to eradicate pathogens. As a result, cell division and cell cycle regulators can be found in the analysis. Both CDC25A and CCNA2 regulate cell cycle transition in G1/S phase [141]. Cell cycle control during G1/S phase is a critical point for cell division. Hence, drug repurposing targeting these regulators could improve the excessive leukocyte proliferation in the cytokine storm, leading to decreased morbidity and mortality rate in severe COVID-19.

**Figure 8:**
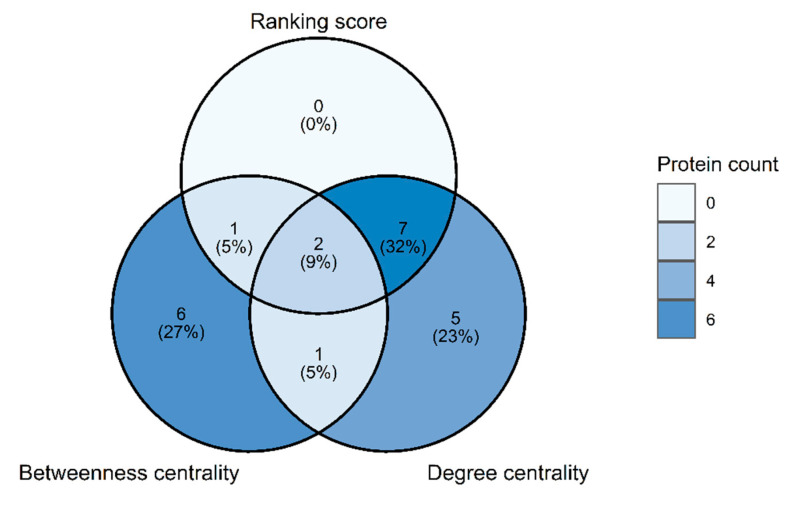
Venn diagram of the key proteins in the ranking score, degree, and betweenness centrality.

To compare with the conventional methods usually used for identifying key proteins in IPINs such as degree and betweenness centrality, Figure 8 displays a Venn diagram of nodes found in the ranking score, degree, and betweenness centrality. The figure shows that the score covers mostly proteins in degree centrality (9 of 15 nodes in the degree set). Conversely, the score captured a few nodes in betweenness centrality (3 of 10 nodes in the betweenness set). Noticeably, the nodes merging between degree and betweenness set cover almost the high-value nodes analyzed from the four centralities (22 of 23 nodes). Thus, a combination of degree and betweenness centrality can provide the best result for identifying key proteins in IPINs. Although the ranking score could not capture other key proteins different from these two centralities, RSAD2 and IFIT1 (the top two genes in Table 4) were detected as the most important immune-related proteins.

RSAD2 or viperin is a broad-spectrum antiviral protein in several viruses such as measles, coxsackievirus A16, and enterovirus A71 [142,143,144]. Moreover, an animal study revealed that RSAD2 was necessary for dendritic cell development [145]. The DEGs analysis of the overlapping genes in postmortem lung tissue from COVID-19 cases and acute lung injury (ALI) murine model found that RSAD2 had a high degree centrality in a COVID-19-associated regulatory network [146]. Moreover, a lower respiratory tract transcriptomic study revealed that *RSAD2* expression correlated with the viral load in mild and severe COVID-19 [147]. IFIT1 is an antiviral protein interacting with other IFIT family proteins to form an IFN-dependent multiprotein complex. The complex plays an important role to increase innate immunity against RNA viruses via binding between IFIT1 and 5′-triphosphate RNA (PPP-RNA) [148]. Nevertheless, an experimental finding revealed that several SARS-CoV-2 proteins, such as nsp7, nsp15, M, 3CLpro, helicase, and N proteins, suppressed *IFT1* expression in HEK293T cells [149].

Although the ranking score was computed to find the key proteins by considering the four centralities, it captured a few proteins when compared with the combination between degree and betweenness centrality. Moreover, the score covered the proteins mostly found in degree centrality. The reason to describe the result is that the proteins with high eigenvector centrality were the subset in degree centrality. Meanwhile, some proteins with high closeness centrality were found in both eigenvector and degree centrality. In addition, proteins found in betweenness centrality rarely overlapped with degree centrality. Therefore, the score calculation is weighted to degree centrality more than betweenness centrality. Noticeably, the combination of degree and betweenness centrality covered almost high-value centrality proteins rather than the ranking score, although it lost one protein with a high closeness value (CCNE1).

As described earlier, the 23 key proteins identified using centrality measurement were involved in innate immune response, cell cycle regulation, and apoptosis. IFI35 is an IFN signaling regulator response to viral infections. Many studies have found that IFI35 plays an essential role in cytokine storms and severity in COVID-19 and influenza [150,151,152,153]. IFIH1 is an intracellular viral sensing protein that stimulates the IFN signaling pathway when viral particles are detected in the cell [154]. IFIH1 was reported to participate in SARS-CoV-2 sensing and was associated with proinflammatory cytokine overproduction in COVID-19 [155,156,157]. DDX58 also plays a role as a cytoplasmic viral sensor. High *DDX58* expression in COVID-19 was associated with cytokine responses [158]. IFI6, IFIT1-3, IRF7, ISG15, MX1, OAS1, OAS2, OASL, and RSAD2 have antiviral activity functions. Many studies in IFI6 have revealed that IFI6 plays an essential role against hepatitis B virus (HBV) and flavivirus replication [159,160]. Several systems biological and transcriptomic studies have also shown that IFI6 is a hub gene in the gene co-expression networks and transcriptomic profiles in COVID-19 [52,161,162,163]. *IFIT1-3*, *OAS1-3*, and *OASL* were upregulated in SARS-CoV-2 and other coronavirus infections [164,165,166,167]. Furthermore, the *IRF7* mutation causing loss of function was reported to be associated with severe pneumonia progression in COVID-19 [168,169]. The infected macrophage cell line study showed that extracellular ISG15 stimulated proinflammatory cytokine production, leading to hyperinflammation [170]. The result of a COVID-19 case-control study revealed that *MX1* expression was increased depending on elevated viral load, and the expression was decreased in elderly patients [171]. Older patients have a high risk for COVID-19-associated cytokine storms, suggesting that low *MX1* expression could play a vital role in the cytokine storm. STAT1 is a signal transduction protein related to various signaling pathways such as IFN, IL-6, epidermal growth factor (EGF), and platelet-derived growth factor (PDGF) pathways [172,173,174]. Several studies have indicated that phosphorylated STAT1 increases in severe COVID-19 patients, causing STAT1 signaling dysfunction and failed IFN activation [175,176,177]. XAF1 is a tumor suppressor protein playing as a positive feedback regulator in the p53-induced apoptotic signaling pathway [178]. Numerous studies have reported that XAF1 dysfunction plays a vital role in tumor progression [179,180,181,182]. A single-cell transcriptomic study in peripheral blood mononuclear cells showed that COVID-19 caused XAF1-induced T lymphocyte apoptosis, leading to adaptive immune impairment [183]. In addition, IPIN analysis from COVID-19 patient lung tissue revealed that IFIH1, DDX58, ISG15, OASL, and XAF1 were hub genes in the network [184].

CCNA2 and CCNE1 are CDK kinase regulators during G1/S and G2/M phases in the cell cycle. Numerous studies have indicated CCNA2 and CCNE1 play a central role in various types of malignancy such as hepatocellular carcinoma, breast, and colon cancer [185,186,187,188]. CDC25A is a protein required in the cell cycle by activating CDKs [189]. *CDC25A* overexpression was found in head and neck, breast, ovarian, and non-small cell lung cancer [190,191,192,193]. An immune study revealed that CDC25A had activities decreasing IFN-β transcription and DDX58-mediated antiviral signaling pathway [194]. CDC20 has a function involved in chromosome segregation and is the target for spindle assembly checkpoint (SAC) [195,196]. High-expressed *CDC20* was related to the worst prognosis in lung squamous cell carcinoma [197]. An IPIN analysis in COVID-19-induced thrombocytopenia also reported that *CDC20* was highly expressed in COVID-19 with thrombocytopenia [198]. CMPK2 is an enzyme associated with the nucleotide salvage pathway. Many studies have shown that CMPK2 participates in IFN-I activation and antiviral immune response [199,200,201]. In COVID-19 studies, *CMPK2* was highly upregulated in severe cases related to ARDS [202,203]. Moreover, FOXM1-dependent tissue regeneration is impaired in severe COVID-19 cases, causing sustained lung injury and a high fatality rate [204]. RRM2, a cell cycle regulator, had an increased expression in lung adenocarcinoma with a poor prognosis [205]. A gene co-expression network and functional enrichment study also showed that RRM2 was a component in a module involved in p53 signaling pathway, a cell cycle and apoptosis pathway, in COVID-19 [206].

### 3.4. Potential Drugs to Cure Severe COVID-19 Patients

We used the key immune-related proteins from the ranking score, degree, and betweenness centrality to find the drug–gene or drug–protein interactions from four well-known public databases: DrugBank database [110], TTD [111], CTD [112], and GeneCards [113]. In addition, the protein not found in both degree and betweenness centrality (CCNE1) was used to discover the interaction. The result from database searching revealed 115 FDA-approved drugs interacting with the key genes or proteins. Table S13 in Supplementary Materials shows the drug–gene and drug–protein interactions in detail among the 23 key immune-related proteins.

**Figure 9:**
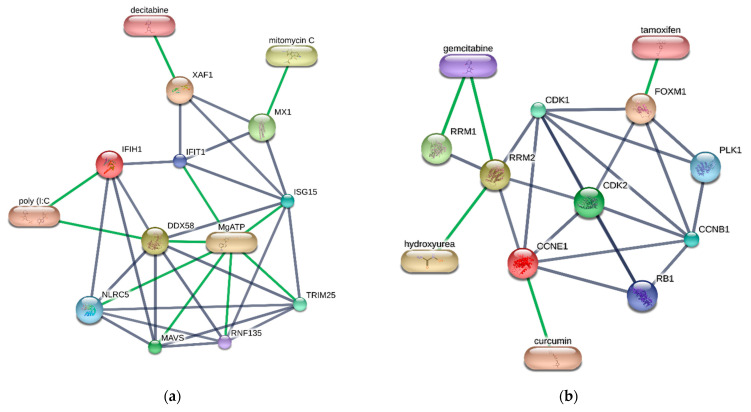
Drug–protein interaction network of candidate drugs targeting the key proteins resulted from STITCH v5.0. (**a**) Drug–proteins interaction network involved in innate immune response and apoptosis. (**b**) Drug–proteins interaction network involved in cell cycle regulation. Grey and green edges represent protein–protein and drug–protein interactions, respectively.

STITCH v5.0 [114], a drug–protein interaction database, was conducted to confirm the result from the databases by the confidence score cut-off value of 0.9. The STITCH result is demonstrated in Figure 9. The key immune-related proteins are classified into two groups. The former is an antiviral, innate immune response, and apoptosis signaling pathway group, and the latter is a cell division and cell cycle group. Figure 9a displays the drug–protein interaction network in the innate immune response, and Figure 9b illustrates the drug–protein interaction network involved in cell cycle regulation. There are seven candidate drugs interacting with these seven key proteins. Three drugs are associated with the key proteins related to innate immune response and apoptosis. In contrast, the rest interacts with the proteins involved in cell cycle regulation. In the innate immune and apoptosis network, polyinosinic:polycytidylic acid (poly(I:C)) interacts with IFIH1 and DDX58, while mitomycin C interacts with MX1. Decitabine also binds to XAF1 in the network. There are four drug–protein interactions in the cell cycle regulation network. RRM2 is interacted with gemcitabine and hydroxyurea, respectively. Tamoxifen binds to FOXM1, and curcumin has an interaction with CCNE1.

From the STITCH v5.0 result, the seven candidate drugs interacted with the seven key proteins. For instance, Poly(I:C) interacted with IFIH1 and DDX58. Poly(I:C) is an immune stimulant used to activate innate immunity such as IFN by the TL3 agonist effect. It also induces cancer cell apoptosis in various types of malignancy: cervical, prostate, and colon cancer [207,208,209,210,211]. Poly(I:C) also increases cytotoxic activity in CD4+ T lymphocytes in viral infections, promoting adaptive immune response [212]. A study in influenza A virus (IAV) and a SARS-CoV-infected mice model revealed that poly(I:C) had a protective effect in fatal respiratory infections [213]. Interestingly, intranasal poly(I:C) in mice with SARS-CoV-2 infection showed a decreased viral load, suggesting that poly(I:C) can be an effective drug for treating the disease [214]. However, some studies reported that poly(I:C) increased proinflammatory production [215,216,217]. Therefore, further studies about poly(I:C) in COVID-19 treatment should consider the drug dosage and administration route to prevent the cytokine storm due to the medication. Mitomycin C, a chemotherapeutic agent, interacted with MX1. It is used to treat many cancer types [218,219,220,221]. A systems biological study revealed that mitomycin C interacted with MX1, a key protein in an IPIN, suggesting further studies in the role of the drug in antiviral stimulation [50]. In addition, there was a drug–protein interaction between decitabine and XAF1. Decitabine is a pyrimidine nucleoside antimetabolite used to treat myelodysplastic syndrome (MDS) and acute myeloid leukemia (AML) [222,223]. A study in the mice model showed that decitabine improved FOXM1-dependent endothelial regeneration and vascular repair [204]. As mentioned above, lung tissue degeneration can cause worsening lung injury. Decitabine then could play a role in decreasing lung injury in severe COVID-19. Interestingly, there is a clinical trial that has been studying decitabine treatment in critical ill COVID-19 patients. The estimated research completion date August 2022. Gemcitabine and hydroxyurea also interacted with RRM2. Gemcitabine, a pyrimidine nucleoside analog and chemotherapeutic agent, is used to treat solid tumors such as bladder, pancreatic, breast, and non-small cell lung cancer [224]. Several studies in cell lines have found that decitabine decreases SARS-CoV-2 replication [225,226,227]. In addition, in a cohort study, gemcitabine reduced SARS-CoV-2 infection in cancer patients [228]. Hydroxyurea, an antimetabolite treating sickle cell anemia, has anti-inflammatory and immunomodulatory effects and was expected to apply well in COVID-19 [229].

A clinical study also revealed that the mortality rate was low in COVID-19 patients receiving hydroxyurea, suggesting a vital role of hydroxyurea in COVID-19 treatment [230]. Tamoxifen, a selective estrogen receptor modulator, had an interaction with CCNE1. The drug is used to treat estrogen receptor-positive breast cancer [231]. Tamoxifen downregulates TMPRSS2, preventing SARS-CoV-2 entry into host cells [232]. A preclinical study showed that tamoxifen reduced SARS-CoV-2 in vitro and in vivo [233]. Moreover, a review article in clinical studies found that tamoxifen decreased COVID-19 susceptibility rates in breast cancer patients [234]. Tamoxifen also inhibited viral replication and virus entry in many virus types such as EBOLA, MERS, and SARS-CoV-2 [234]. The drug repurposing result also revealed that curcumin interacted with CCNE1. It is worth noting that curcumin is a promiscuous molecule acting on many receptors. Hence, the effect of curcumin in COVID-19 can be from other mechanisms. Our study only proposed one of the possible mechanisms of curcumin in severe COVID-19 treatment. Curcumin is a natural product found in turmeric (*Curcuma longa*). Many studies have indicated that curcumin has anti-inflammatory and antioxidant effects [235,236,237]. Moreover, it provides effects in apoptosis promotion, cell proliferation inhibition, anti-cell adhesion and invasion, decreased angiogenesis, and anti-microbial activity [238]. Therefore, its clinical application is related to numerous diseases, such as rheumatoid arthritis, inflammatory bowel diseases, osteoarthritis, and various types of cancer. In COVID-19, several review articles have revealed that curcumin inhibits viral entry and replication [239,240,241]. It also promotes IFN and antiviral signaling pathway and decreases proinflammatory cytokine production. Curcumin has protective effects on ARDS by reducing NF-κB, inflammasome, and IL-8 pathway. Furthermore, a randomized control trial study showed that mild, moderate, and severe COVID-19 patients taking oral curcumin with piperine had better clinical outcomes and lower hospitalization duration than the controls [242]. A systematic review reported that curcumin reduced the proinflammatory cytokines, such as IL1β and IL6. It also increased the anti-inflammatory cytokines, for example, IL-10, IL-35 and transforming growth factor α (TGF-α) [243]. Therefore, further study should focus on the effective dose and administration routes of curcumin.

## 4. Discussion

This study constructed the immune-related protein interactions network, IPIN, for severe COVID-19 based on the leukocyte transcriptomic profile of critically ill patients using network propagation on the human interactome network. Functional enrichment analysis and network clustering were operated to discover the underlying molecular mechanisms of immune-related severe COVID-19. Topological analysis, centrality, and ranking score measurements were calculated to identify the key immune-related proteins. Finally, the drug–protein interactions were searched to find the candidate drugs to treat the severe COVID-19 patients.

Diffusion-based IPIN construction and permutation testing provided the highly connected immune-related proteins in IPIN. IPIN was a network with a small-world effect in relation to other biological networks. The small-world effect was proved by the low average shortest path length and high average clustering coefficient. The scale-free property cannot be explained in the network due to a lack of a relationship between degree and its probability. However, the IPIN was less likely to be the random network because it provided the significant PPI enrichment p-value from the STRING database. We performed the four network centralities (degree, betweenness, closeness, and eigenvector) and ranking score to find the key immune-related proteins. The results showed that the combination of degree and betweenness centrality covered a wide range of the key proteins. However, the ranking score can detect the main key proteins: RSAD2 and IFTI1.

We identified 23 key immune-related proteins, such as CCNA2, CCNE1, CDC20, CDC25A, CMPK2, DDX58, FOXM1, IFI6, IFI35, IFIH1, IFIT1, IFIT2, IFIT3, IRF7, ISG15, MX1, OAS1, OAS2, OASL, RRM2, RSAD2, STAT1, and XAF1, using the four centralities and ranking score measurement. These proteins all play an important role in severe COVID-19, validated by several computational, experimental, and clinical studies. The functional enrichment analysis from the whole network and the modules obtained from both MCODE and MCL methods produced similar results. The enrichment terms were divided into four main categories: cell cycle regulation, antiviral and innate immune response, apoptosis, and nucleotide metabolism. These terms were in accordance with leukocytes during viral infections. Furthermore, the main terms were accepted with the functional classification in the key proteins. For instance, the key proteins involved in cell cycle regulation consisted of CCNA2, CCNE1, CDC20, CDC25A, and RRM2, while the others associated with antiviral and innate immune response were DDX58, FOXM1, IFI6, IFI35, IFIH1, IFIT1, IFIT2, IFIT3, IRF7, ISG15, MX1, OAS1, OAS2, OASL, RSAD2, and STAT1. In addition, the remaining key proteins such as CMPK2 and XAF1 play a crucial role in nucleotide metabolism and apoptosis, respectively.

Drug repurposing based on drug–gene and drug–protein interaction database searching provided the seven potential candidate drugs, poly(I:C), mitomycin C, decitabine, gemcitabine, hydroxyurea, tamoxifen, and curcumin. There were three drugs interacting with the key proteins related to antiviral and innate immune response: poly(I:C), mitomycin C, and decitabine. Other drugs interacted with the key proteins involved in cell cycle regulation and apoptosis. Among the candidate drugs, we recommend that the drugs interacting with proteins involved in IFN and antiviral signaling should be used carefully in clinical application because they promote IFN stimulation. IFN overactivation can result in excessive proinflammatory cytokine production in some studies that we mentioned [128,129,130,131]. Moreover, chemotherapeutic agents cause many adverse side effects and should be performed as the second choice. Therefore, we suggest using curcumin and tamoxifen as the first choices for clinical application. They have fewer side effects than other chemotherapeutic agents because curcumin is a natural product and tamoxifen is a targeted drug. In addition, both curcumin and tamoxifen have several clinical studies to support their effectiveness in COVID-19 treatment.

## 5. Limitations and Future Study Suggestions

Although this study provides novel knowledge and candidate targeted drugs in COVID-19, it has some limitations to be explained. First, the network diffusion method is an algorithm consuming computational time and memory space which depends on the number of nodes, interactions, and permutation tests. We conducted high-performance computing (HPC) for running the LHD algorithm in the original and 1000 random sets to perform the permutation test. Second, many proinflammatory cytokine signaling pathways, such as IL-1β, IL-6, IL-12, IL-18, IL-33, and TNF-α, were rarely detected in this IPIN analysis. Our reason for explaining the issue is that the data came from comparing controls and COVID-19 cases in an intensive care unit. Typically, critically ill patients have stress and inflammatory responses, leading to increased proinflammatory cytokines. Therefore, there was no difference in the proinflammatory cytokine gene expression between cases and controls. Moreover, proinflammatory cytokines are usually released from respiratory epithelial and immune cells in the lung parenchymal tissue. Studies in peripheral white blood cells can lose this proinflammatory cytokine information. Our suggestion for future research is to perform lung transcriptomic profiles comparing severe COVID-19 cases and mild illness or healthy cases for IPIN construction and analysis. Furthermore, a single-cell approach should be conducted to identify an IPIN in each cell type. In COVID-19, there are differences between each cell type such as cell count, behavior, function, and pathogenesis. Therefore, identifying key proteins in these cells can help to treat the disease more precisely.

## 6. Conclusions

This study proposed LHD algorithms to perform network diffusion on the human interactome network to construct the immune-related IPIN in severe COVID-19 based on the transcriptomic data. Functional enrichment analysis found that the network contained the proteins involved in antiviral and innate immune response signaling pathways, cell cycle regulation, apoptosis, and protein processing. The degree and betweenness centrality combination covered almost the key proteins from the four centrality measurements. These key proteins play a significant role in cell proliferation, antiviral activity, and innate immunity responding to the SARS-CoV-2 infection. Moreover, the candidate drugs targeting the key proteins were found from database searching. Most of them have experimental data supporting their effectiveness in COVID-19 treatment. However, other key proteins and candidate drugs were not found in our method and need further investigation. Therefore, a combination of advanced experimental and computational tools should be conducted for further efficient treatment discovery relating to COVID-19.

## Figures and Tables

**Table 1:** Global Topological Parameters of the IPIN.

Symbol	Description	Value
*N*	Number of nodes	97
*M*	Number of edges	778
<*k*>	Average degree	16.04
*d*	Diameter	7
*r*	Radius	4
*mspl*	Mean shortest path length	3.01
*D*	Density	0.17
*acc*	Average clustering coefficient	0.74

**Table 2:** Clustering of functional enrichment analysis in the IPIN by Molecular Complex Detection (MCODE) algorithm.

Functional Component	Term ID	Biological Term	Log10 (*p*-Value)
MCODE1	R-HSA-909733	Interferon alpha/beta signaling	−70.8
	R-HSA-913531	Interferon signaling	−57.5
	R-HSA-1280215	Cytokine signaling in Immune system	−42.5
MCODE2	R-HSA-2467813	Separation of sister chromatids	−13.6
	R-HSA-69278	Cell cycle, mitotic	−13.5
	GO:0098813	Nuclear chromosome segregation	−13.0
MCODE3	R-HSA-69615	G1/S DNA damage checkpoints	−12.0
	R-HSA-176409	APC/C:CDC20 mediated degradation of mitotic proteins	−11.7
	R-HSA-176814	Activation of APC/C and APC/C:CDC20 mediated degradation of mitotic proteins	−11.7
MCODE4	R-HSA-983168	Antigen processing: ubiquitination and proteasome degradation	−6.0
	R-HSA-983169	Class I MHC mediated antigen processing and presentation	−5.7
	GO:0016567	Protein ubiquitination	−5.0

**Table 3:** Summary of biological function of 23 nodes with high centrality predominance.

Symbol	Description	High Centrality	Biological Function
CCNA2	Cyclin A2	DC, BC, CC	Cell cycle regulation
CCNE1	Cyclin E1	CC	Cell cycle regulation
CDC20	Cell Division Cycle 20	BC	Cell cycle regulation
CDC25A	Cell Division Cycle 25A	BC, CC	Cell cycle regulation
CMPK2	Cytidine/Uridine Monophosphate Kinase 2	BC, CC	Salvage nucleotide synthesis
DDX58	DExD/H-Box Helicase 58	BC, CC	Viral dsRNA recognition
FOXM1	Forkhead Box M1	BC	Transcription activator in cell proliferation
IFI6	IFN-α Inducible Protein 6	DC, EC	Apoptosis regulation and antiviral activity
IFI35	IFN Induced Protein 35	DC, BC	Regulation of innate immune signaling parhway
IFIH1	IFN Induced With Helicase C Domain 1	BC, CC	Intracellular sensor of viral RNA
IFIT1	IFN Induced Protein With Tetratricopeptide Repeats 1	DC, CC, EC	Viral replication inhibition
IFIT2	IFN Induced Protein With Tetratricopeptide Repeats 2	DC, EC	Viral replication inhibition
IFIT3	IFN Induced Protein With Tetratricopeptide Repeats 3	DC, EC	Viral replication inhibition
IRF7	IFN Regulatory Factor 7	DC, EC	Antiviral activity
ISG15	IFN-stimulated gene 15	DC, CC, EC	Antiviral activity
MX1	MX Dynamin Like GTPase 1	DC, CC, EC	Viral replication inhibition
OAS1	2′-5′-Oligoadenylate Synthetase 1	DC	Viral replication inhibition
OAS2	2′-5′-Oligoadenylate Synthetase 2	DC	Viral replication inhibition
OASL	2′-5′-Oligoadenylate Synthetase Like	DC	Antiviral activity
RRM2	Ribonucleotide Reductase Regulatory Subunit M2	BC, CC	Cell cycle regulation
RSAD2	Radical S-Adenosyl Methionine Domain Containing 2	DC, BC, CC, EC	Antiviral activity
STAT1	Signal Transducer And Activator Of Transcription 1	DC, EC	Stimulation of proinflammatory cytokines
XAF1	XIAP Associated Factor 1	DC, EC	Antiapoptotic inhibition

IFN, interferon; XIAP, X-linked inhibitor of apoptosis protein; dsRNA, double-strand RNA; DC, degree centrality; BC, betweenness centrality; CC, closeness centrality; EC, eigenvector centrality.

**Table 4:** List of nodes with high-ranking scores.

Ensembl ID	Symbol	Ranking Score
ENSP00000371471	RSAD2	4.166667 × 10^−2^
ENSP00000360869	IFIT1	4.629630 × 10^−3^
ENSP00000368699	ISG15	6.666667 × 10^−4^
ENSP00000381601	MX1	4.370629 × 10^−4^
ENSP00000303706	CDC25A	1.940994 × 10^−4^
ENSP00000360883	IFIT3	1.449275 × 10^−4^
ENSP00000354394	STAT1	1.017501 × 10^−4^
ENSP00000274026	CCNA2	6.410256 × 10^−5^
ENSP00000360891	IFIT2	4.409171 × 10^−5^
ENSP00000380697	IRF7	4.084967 × 10^−5^

## Data Availability

The data generated in this study are available in this article.

## Supplementary Materials

The following supporting information can be found and downloaded at: https://www.mdpi.com/article/10.3390/biom12050690/s1. Table S1: 224 differentially expressed genes (DEGs) (FDR<0.05 and log2 FC > 1.5); Table S2: The DEGs in the protein-protein interaction network from STRING with Ensembl protein IDs; Table S3: The immune-related proteins from DEGs in the human interactome data as seed nodes for network diffusion; Table S4: Diffusion scores of the original set and statistic data of each node in the whole PPI network; Table S5: 154 protein nodes with significant diffusion scores by the permutation test; Table S6: Degree, betweenness, closeness, and eigenvector centrality values of each node in the IPIN; Table S7: Network clustering of the IPIN by MCL algorithm provided 4 modules: MCL1, MCL2, MCL3, and MCL4. Table S8: The detail of enrichment results in each MCL module; Table S9: List of nodes with high degree centrality; Table S10: List of nodes with high betweenness centrality; Table S11: List of nodes with high closeness centrality; Table S12: List of nodes with high eigenvector centrality; Table S13: Drug-gene interactions of the identified key immune-related proteins; Table S14: Edgelist of the IPIN.
