# Supplementary material for: Immune-Related Protein Interaction Network in Severe COVID-19 Patients toward the Identification of Key Proteins and Drug Repurposing
Source: Biomolecules. 2022 May 11;12(5):690. doi: 10.3390/biom12050690 (PMC9138873; doi:10.3390/biom12050690)
Supplement: Supplementary file 1 [file biomolecules-12-00690-s001.zip › Supplementray Table S9-S12.pdf]

**Table S9.** List of nodes with high degree centrality.

| Ensembl ID | Symbol | Degree Centrality |
|---|---|---|
| ENSP00000360869 | IFIT1 | 39 |
| ENSP00000368699 | ISG15 | 38 |
| ENSP00000371471 | RSAD2 | 38 |
| ENSP00000381601 | MX1 | 38 |
| ENSP00000360883 | IFIT3 | 37 |
| ENSP00000354394 | STAT1 | 36 |
| ENSP00000360891 | IFIT2 | 34 |
| ENSP00000380697 | IRF7 | 33 |
| ENSP00000257570 | OASL | 32 |
| ENSP00000274026 | CCNA2 | 31 |
| ENSP00000342278 | OAS2 | 31 |
| ENSP00000342513 | IFI6 | 31 |
| ENSP00000354822 | XAF1 | 31 |
| ENSP00000388001 | OAS1 | 31 |
| ENSP00000395590 | IFI35 | 31 |

**Table S10.** List of nodes with high betweenness centrality.

| Ensembl ID | Symbol | Betweenness Centrality |
|---|---|---|
| ENSP00000303706 | CDC25A | 0.248509 |
| ENSP00000371471 | RSAD2 | 0.221581 |
| ENSP00000256722 | CMPK2 | 0.207767 |
| ENSP00000353770 | RRM2 | 0.205415 |
| ENSP00000274026 | CCNA2 | 0.186888 |
| ENSP00000263642 | IFIH1 | 0.149838 |
| ENSP00000369213 | DDX58 | 0.111075 |
| ENSP00000361540 | CDC20 | 0.051103 |
| ENSP00000395590 | IFI35 | 0.042906 |
| ENSP00000342307 | FOXM1 | 0.041747 |

**Table S11.** List of nodes with high closeness centrality.

| Ensembl ID | Symbol | Closeness Centrality |
|---|---|---|
| ENSP00000371471 | RSAD2 | 0.436364 |
| ENSP00000303706 | CDC25A | 0.428571 |
| ENSP00000256722 | CMPK2 | 0.424779 |
| ENSP00000353770 | RRM2 | 0.422907 |
| ENSP00000263642 | IFIH1 | 0.419214 |
| ENSP00000274026 | CCNA2 | 0.417391 |
| ENSP00000369213 | DDX58 | 0.415584 |
| ENSP00000262643 | CCNE1 | 0.388664 |
| ENSP00000360869 | IFIT1 | 0.38247 |
| ENSP00000368699 | ISG15 | 0.380952 |
| ENSP00000381601 | MX1 | 0.380952 |

**Table S12.** List of nodes with high eigenvector centrality.

| Ensembl ID | Symbol | Eigenvector Centrality |
|---|---|---|
| ENSP00000360869 | IFIT1 | 1 |
| ENSP00000381601 | MX1 | 0.994698 |
| ENSP00000368699 | ISG15 | 0.994698 |
| ENSP00000371471 | RSAD2 | 0.989394 |
| ENSP00000360883 | IFIT3 | 0.985845 |
| ENSP00000360891 | IFIT2 | 0.968306 |
| ENSP00000354394 | STAT1 | 0.963544 |
| ENSP00000354822 | XAF1 | 0.93284 |
| ENSP00000342513 | IFI6 | 0.93284 |
| ENSP00000380697 | IRF7 | 0.927411 |
